# Consumer Preferences for Traceable Food with Different Functions of Safety Information Attributes: Evidence from a Menu-Based Choice Experiment in China

**DOI:** 10.3390/ijerph17010146

**Published:** 2019-12-24

**Authors:** Bo Hou, Jing Hou, Linhai Wu

**Affiliations:** 1 School of Philosophy and Public Administration, Jiangsu Normal University, Xuzhou 221116, China; houbo@jsnu.edu.cn; 2 School of Business, Jiangsu Normal University, Xuzhou 221116, China; houjing@jsnu.edu.cn; 3 Food Safety Research Base of Jiangsu Province (School of Business), Jiangnan University, Wuxi 214122, China

**Keywords:** food safety, foodborne diseases, ex ante quality verification, ex post traceability, menu-based choice experiment

## Abstract

It is of great value to study consumer demand for safe foods in promoting the development of a safe food market system and the reduction of food safety risks, as well as foodborne diseases in China. This paper takes traceable pork as an example and constructs food safety information attributes with ex ante quality verification and ex post traceability. Interactions between safety information attributes and the consumer’s response to cost-driven price changes were investigated for 345 consumers in Wuxi City, Jiangsu Province, China, using a menu-based choice (MBC) experiment and multivariate probit (MVP) model as analysis tools. The results suggest that food safety information attributes are important to consumers, as the consumers preferred pork quality inspection attributes to pre-incident quality assurance functions. Therefore, it is beneficial to include pork quality inspection attributes in the traceable pork attribute systems during the initial construction of traceable pork markets in China. Attribute price was an important factor that affected consumers’ choice of information attributes. When customization cannot be achieved, a profile composed of elastic pork quality inspection attribute and supply chain–internal traceability attribute would be the most preferred traceable pork product in the market based on the need of building a fully functional traceable food system and reducing food safety risks. In addition, there was a strong substitution relationship between different information attributes, and there was heterogeneity in consumers’ choice of information attributes. Therefore, the government should support manufacturers in producing multi-level safe food to meet diverse consumer demand.

## 1. Introduction

Frequent food safety problems in recent years have continuously and repeatedly unsettled the Chinese public. In fact, food safety risks are a common problem facing many countries worldwide. Consumers worldwide are often faced with varying degrees of food safety risks. According to the World Health Organization (WHO) estimates, approximately 2.2 million deaths are caused by foodborne or watery diarrhea every year throughout the world [1]. Approximately 5000 people died each year in the United States prior to 1999 from foodborne disease [2]. Since 2011, the total number of foodborne disease cases has been estimated to be 48 million in the United States, with 3000 annual deaths [1]. The situation in China is even worse. Many food safety incidents have occurred in China, such as tainted melamine milk powder, Sudan red salted duck eggs, and sick or dead pigs in the market; these incidents have caused more than 50,000 people to become sick or die [3].

When consumers’ health is impaired, they often cannot immediately or definitively attribute the disease to a certain food. Moreover, consumers cannot observe the production process, thus leading to information asymmetry in food safety information attributes [4]. Food traceability systems are able to monitor food production and distribution by generating a reliable continuous flow of safety information in the supply chain, to identify the source of the problem, and recall related products through traceability [5]. These systems are, therefore, considered a major tool for the effective elimination of information asymmetry and the fundamental prevention of food safety risks [6] and have been widely implemented in America as well as many European countries. China has continued to promote the construction of food traceability systems since 2010. However, the construction of food traceability systems has not fundamentally reduced food safety incidents [7].

Regulation (EC) No. 178/2002, implemented by the European Union, strictly defines the content of food traceability information, requiring complete traceability information covering all processes of the food supply chain, and outlines the information attributes required for each process. Hobbs [4] indicated that complete food traceability systems should possess the basic functions of both ex ante quality verification and ex post traceability. Ex ante quality verification enables consumers to confirm food safety and quality prior to purchase via credence attribute labels. Ex post traceability allows for timely recall with complete traceability information along the food supply chain in the event of food safety problems and establishes accountability for such problems. Currently, the policies of the Chinese traceable pork market system mainly focus on sporadic work guidelines and pilot traceable pork systems in the Chinese market, which only allow for ex post traceability [8] and might explain the current failure of such systems [9]. The production of traceable pork with both ex ante quality verification and ex post traceability will inevitably increase costs, which will be reflected in the market price. Since consumers might not be willing to pay (WTP) such an increased price, investigating the market demand for traceable pork with ex ante quality verification and ex post traceability will positively contribute to the development of a traceable food market system and the reduction of foodborne diseases in China.

Compared with ordinary food, traceable food is composed of traceability, transparency, and quality assurance [10]. Hobbs [4] summarized the effects of establishing food traceability systems into two basic functions: ex ante quality verification and ex post traceability. Research shows that a combination of animal welfare, origin certification, quality inspection, environmental impact, safety assurance, and other information attributes in food traceability systems can provide ex ante quality verification, which can play a greater role than ex post traceability in eliminating information asymmetry [4,11]. A product can have search, experience, and confidence attributes [12]; ex ante quality verification provides credence attributes related to food safety in the form of labels, which is equivalent to converting credence attributes of food safety to search attributes, thus reducing the search costs [8]. Thus, traceable food can be considered a combination of various information attributes.

Numerous studies have been performed on consumer preferences for traceability information, quality, and safety assurance, as well as other information attributes. For example, Loureiro and Umberger [13] indicated that U.S. consumers showed higher preferences for beef with food safety inspection and certification information from the United States Department of Agriculture than that with traceability only. Furthermore, Morteza [14] suggested that the vast majority of Canadian consumers were willing to pay a 15% premium for certified farmed Atlantic salmon. Lu et al. [15] indicated that Chinese consumers had the highest WTP for government certification information and attached the greatest importance to farming information and comprehensive traceability information on the whole. Relevant studies have attempted to explore the relationships among traceability information attributes while evaluating consumer preferences. Wu et al. [8] revealed a substitute relationship between traceability to slaughter and processing and local production and showed a complementary relationship between traceability to slaughter and processing and nonlocal production. Ortega et al. [16] reported a substitute relationship between government quality and safety inspection and third-party quality certification and between third-party quality certification and traceability, as well as a complementary relationship between government quality and safety inspection and additional information labeling, between additional information labeling and traceability, and between government quality and safety inspection and traceability. Ubilava and Foster [17] found a substitute relationship between government food safety and quality assurance and supply chain traceability. Lim et al. [18] demonstrated a complementary relationship between safety assurance, such as bovine spongiform encephalopathy detection, and traceability.

The existing literature provides a useful reference for research. However, there are still some remaining deficiencies. First, the majority of earlier research made no strict distinction between ex ante quality verification and ex post traceability attributes of traceable food. Moreover, most previously investigated information attributes of traceable food have been related to ex post traceability [19,20] and lacked holistic research on traceable pork attributes. In fact, the information attributes of different functions have different effects on consumer preferences. Second, although substitute or complementary relationships between attributes have been determined, one-way or two-way interrelationships between attributes have not yet been investigated. Furthermore, the amount of information included in the attributes themselves has not been considered (the definition of the amount of information contained in traceability attributes varies between studies. Some studies only cover one or a few processes for traceability, and some cover all processes of the entire supply chain.). In fact, the degree of mutual substitutability can vary between different amounts of information and between two similar attributes. Third, choice experiments, conjoint analyses, and contingent valuation methods remain the major techniques used in most studies. However, these mainstream research methods have flaws, for example, when the profiles of attributes and levers are given, consumers will be forced to choose even if there is a substitutional relation between the attributes [21]. In addition, in order to simulate different scenarios, it is usually necessary to design multiple tasks, each of which has more than two options. Therefore, consumers will have a large reaction error from completing the first task to the last task [22]. Most importantly, consumers are not sensitive to virtual profile prices during the experiment [23]. The purpose of this paper is to explore the interactions between multiple attributes and the consumer’s response to cost-driven prices changes. Therefore, exploring the appropriate tools needed to capture a consumer’s corresponding response to price changes will facilitate more accurate research.

Taking pork as a case, we constructed a relatively complete traceable pork attribute system consisting of supply chain traceability, supply chain–internal traceability, pork quality inspection, and enterprise quality management system certification attributes. According to Hobbs’ [4] classification criteria for ex ante quality verification and ex post traceability systems, the pork quality inspection and enterprise quality management system certification attributes were regarded as attributes of traceable pork with the ex ante quality verification function, while the supply chain traceability and supply chain–internal traceability attributes were regarded as attributes of traceable pork with the ex post traceability function. Then, we employed a menu-based choice (MBC) experiment to examine consumer preferences for the information attributes of traceable food with ex ante quality verification and ex post traceability functions and to examine the food traceability systems that meet the needs of most Chinese consumers, the results of which can provide a reference for the Chinese government to implement a traceable food policy.

## 2. Materials and Methods

### 2.1. Experimental Object Selection

In 2018, China produced 54.04 million tons of pork, accounting for 50% of the world’s pork production. As the most popular meat protein in China, pork is consumed approximately 4.6 times more frequently, on average, than in the rest of the world. However, pork also frequently suffers from safety incidents in China [7]. Therefore, pork can be investigated as a representative case for food safety and traceability in China. Furthermore, taking into account the possible different consumer preferences for different body parts of traceable pork, pork hindquarters (hereinafter referred to as traceable pork) were selected to exclude interference from non-essential elements when investigating consumer preferences for information attributes with ex ante quality verification and ex post traceability.

### 2.2. Experimental Method Selection

Since traceable pork is not popular in China, and the types of traceable pork with different functional attributes discussed in this study belong to virtual traceable pork profiles (which do not yet exist in the current Chinese market), it is difficult to obtain a large amount of actual purchase data. Therefore, it is suitable to study consumers’ stated preferences for traceable foods. The purpose of this paper is to explore the interactions between multiple attributes and consumers’ responses when the cost-driven price changes. The menu-based choice (MBC) experiment method, one of the preferred research methods, is an appropriate tool to capture the corresponding reactions of consumers when the price changes [24]. The menu-based choice experiment method allows consumers to choose product attributes independently and more accurately simulates consumer product purchase situations in the real market based on mass customization [25]. It also measures consumer price sensitivity. Moreover, menu-based experiments generate more virtual product profiles than those of choice experiments or conjoint analyses, thereby helping solve the problem of the substitution effect between attributes [21] and response errors in multitasking [22], which are common in general choice experiments. Therefore, this study used a menu-based choice experiment method to carry out specific research.

### 2.3. Attributes and Levels Settings in Experiments

#### 2.3.1. Post-Incident Traceability Attributes of Traceable Pork

**Figure 1:**
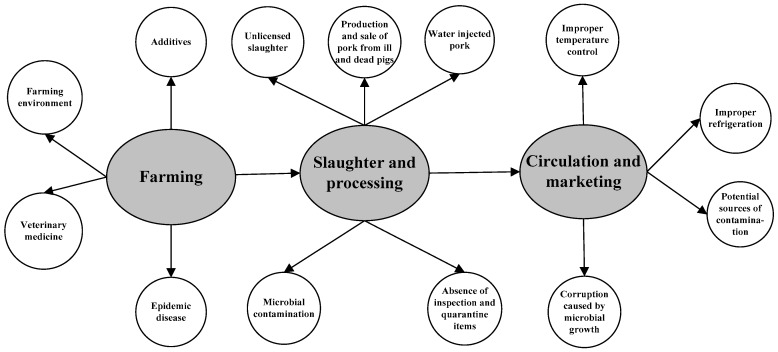
Specific safety risks in the major processes of the pork supply chain.

The effectiveness of ex post traceability depends on whether the establishment of information attributes completely covers the critical risk points in the supply chain. As shown in Figure 1, the main risks to pork are included in all processes of the supply chain system (i.e., farming, slaughter, transportation, and marketing) [9]. A traceability system that includes information attributes of only some of these processes cannot effectively achieve ex post traceability. Moe [26] classified traceability into supply chain traceability and internal traceability, according to the activity or direction in which information is recalled in the food chain. The essence of supply chain traceability is what is commonly referred to as link management in the food supply chain. Internal traceability is the traceability process of the internal production history from food input to output for each process of the supply chain. Therefore, supply chain traceability and supply chain–internal traceability were set as two attributes of the ex post traceability of pork according to the risk processes throughout the pork supply chain and the features of information recalled activities (as shown in Table 1).

#### 2.3.2. Ex Ante Quality Verification Attributes of Traceable Pork

Here we discuss the ex ante quality verification attributes of traceable pork. Chemical and microbiological hazards are considered new challenges in the agricultural alimentary line. European regulations of food safety are dedicated to reducing the risk represented mostly by chemical and microbiological hazards [27]. However, pork inspection and quarantine measures do not include mandatory measurements of the physical, chemical, or microbiological indicators in China. The current outbreak of a series of pork safety incidents has made “clenbuterol” and veterinary drug residues a pork safety risk factor that Chinese consumers are generally concerned about. Therefore, pork quality inspection was set as an attribute, represented by an acceptable product label issued by a third party, to provide consumers with the safety and quality information shown in Table 1. In addition to pork inspection and quarantine, quality management system certification was also set as an attribute for quality assurance, represented by labeling the enterprise with a quality management system certification mark issued by a third party. This reflected the pork quality assurance measures and controllability of the production process [10]. Therefore, based on the reality of China, pork quality inspection and enterprise quality management system certification were set as the two attributes for ex ante quality verification.

#### 2.3.3. Price Levels of Traceable Pork Attributes

The purpose of this paper is to explore the interactions between multiple attributes and the consumer’s response to cost-driven prices changes. Therefore, this study needs to differentiate the price level of attributes to explore the price sensitivity of consumers. The MBC method is an appropriate tool to capture the corresponding reactions of consumers when the price changes [24]. However, the types of traceable pork with different functional attributes discussed in this study do not yet exist in the Chinese market. Therefore, in order to set reasonable price levels for traceable pork with different functional attributes, according to Wu [28], a preliminary willingness to pay experimental auction was conducted for different information attributes using the Becker–DeGroot–Marschak (BDM) mechanism [29] in Wuxi. As such, the consumer-induced value (average price information) in simulated real-world market trading scenarios was obtained. The preliminary experimental auction was conducted by one-on-one interviews in October 2015. A participant won if his/her bid was higher than the computer randomly generated price, then the participant exchanged the corresponding type of safe pork and paid the randomly generated price. If the computer randomly generated price was more than 5 yuan, which was a cash amount compensation for the BDM auction, those who won the auction would pay the remainder out of pocket. Moreover, in order to avoid the response-order effect, auction objects were presented in a random order (see the Appendix A for the preparation and procedure of the BDM experiment). Ultimately, 259 valid samples were obtained in the BDM experimental auction. The results of the BDM experimental auction are shown in Table 2.

According to Orme [24], five price levels are typically set in menu-based experiments. Therefore, based on the BDM experiment results, five price levels were set for pork quality inspection, quality management system certification, supply chain traceability, and supply chain–internal traceability. The mean was taken as the middle price level. The other four price levels were set as the mean ± 0.5 and one standard deviation (see Table 3).

### 2.4. Experimental Task Design

**Figure 2:**
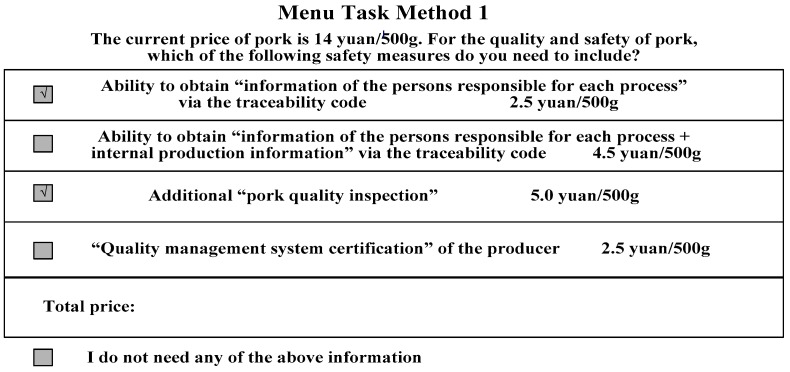
Example of a choice set in the menu-based choice experiment.

In this study, four attributes, each with five price levels, were set. Therefore, 5 × 5 × 5 × 5 = 625 choice profiles were generated using the full factorial design. One choice profile corresponds to each experimental plan in menu-based experiments, and it is unrealistic for participants to choose from 625 × 16 = 10,000 choice sets. In general, participants will become fatigued after distinguishing 15 to 20 choice profiles [8]. It is an inevitable fact that, in order to improve the efficiency of consumer choices, the number of profiles must be reduced. Therefore, it is necessary to optimize the experimental scheme on the basis of satisfying the analytical validity of the data. A fractional factorial design was thus used to design the experimental tasks. According to Orme [30], a questionnaire of 10 versions × 10 menus with the highest design efficiency was generated using Sawtooth MBC 1.0.10 and a balanced overlap randomized design method. In this case, the total number of menus meets the minimum number of tasks required while ensuring the experimental efficiency of the participants. The efficiency test results of the attribute and price level design are shown in Table 4. Efficiency test results indicate that for the first and second price levels of pork quality inspection, quality management system certification, and supply chain traceability, the design frequencies were generally balanced, and the efficiency ratio of the ideal to actual standard deviation was greater than 90%. However, there was more than a 10% difference between the actual and ideal design standard deviations for the third and fourth levels of the supply chain-and-internal traceability and supply chain traceability. This was due to the safety information content of the supply chain-and-internal traceability, including supply chain traceability; moreover, the actual menu tasks were designed using a non-balanced design method, resulting in the price of the supply chain-and-internal traceability being higher than that of supply chain traceability. Figure 2 is an example of the final menu-based choice experimental design.

### 2.5. Implementation of Experiments

There were differences in pork prices and consumer willingness to pay in different regions of China. The experimental city of the MBC experiment, like the BDM experiment, was Wuxi, Jiangsu Province, China to ensure consistency. Wuxi is a leading city in China and also a pilot city for traceable pork. Residents in Wuxi have acquired certain understandings of traceable pork. Therefore, we chose Wuxi to conduct investigations and experiments. If the survey was conducted in a city where a traceability system had not yet been implemented, investigators would have to explain the related concepts in detail. This would not only greatly increase the time costs, but also increase the dependence of the survey results on the investigator’s ability to explain the survey concepts, which could lead to uncertain results. To ensure sample representativeness, participants were randomly recruited by trained investigators in farmer’s markets, supermarkets, and pork stores in five administrative districts of Wuxi (Liangxi, Xishan, Huishan, Hubin, and Xinwu districts). The third consumer that came into view was recruited. Taking into account the shopping habits of residents, the experiment was conducted during the hours of 8:00–10:00 and 16:00–18:00, two periods when most food shopping is done [8]. Prior to the experiment, the experimental auction rules were explained to the participants, and their understanding of the rules was tested. Participants were asked to complete questionnaires at the end of the experimental auction to collect demographics and other data. This experiment was conducted and completed in January 2016. A total of 350 MBC experiments were conducted, among which 345 valid samples were obtained.

### 2.6. Model Construction and Variable Settings

#### 2.6.1. Model Construction

The attribute utility theory of Lancaster was the theoretical basis of the model construction in the menu-based experiments. Lancaster [31] suggested that a consumer’s utility is given by attributes rather than goods. Pork profiles composed of traceability attributes are often used as the basis for constructing utility functions in related studies. Here, the latent utility was obtained by participant n choosing information attribute i in menu option space C under situation t:

(1) $$U_{nit}=\alpha_{ni}+\beta_{ni}^{\prime }X_{nit}+\varepsilon_{nit}$$

where $\alpha_{ni}$ is the constant term, $X_{nit}$ is the vector of attribute price, $\beta_{ni}^{}$ is the parameter estimate, and $\varepsilon_{nit}$ is the stochastic term. Although $U_{nit}$ cannot be observed, it can be discriminated by the choice of participants. When facing each of the safe information attributes and price options on the menu, consumers need to make decisions between choice and no choice. With $Y_{nit}$ as the indicator variable, the following binary choice model was constructed:

(2) $$\left\{ \begin{array}{l} Y_{nit}=1ifU_{nit}>0\\ Y_{nit}=0ifU_{nit}\leq 0 \end{array}\right.$$

Equation (2) indicates that if $U_{nit}>0$, then the participant will choose attribute *i* in period *t* ($Y_{nit}=1$); otherwise, the opposite is true.

Since the MBC method shows four attributes in the experiment tasks, the consumer will select multiple information attributes of the traceable pork at the same time based on their own needs. That is to say, participant *n* needs to make a decision continuously on whether or not to select an attribute of the *i* type. These decisions are not completely mutually exclusive. Therefore, some unobservable factors may cause consumers to simultaneously select different information attributes of traceable pork; that is, the error terms of the four binary probit models are related. If we do not consider the endogeneity problem between the models and only use four simple binary probit models to study the consumer’s decision-making behaviors, the estimation results may be biased. Accordingly, this study employed a multivariate probit (MVP) model [32] as an analytical tool to allow correlation between the error terms of different equations. Furthermore, a number of papers [33,34] have advocated multivariate probit analysis for menu-based problems.

Furthermore, according to Xue [35], and Wu [9,28], consumer’s choice of food attribute is affected by the price of this attribute, the price of other attributes, individual characteristics of the participants, family characteristics, cognitive attitudes, past experience, and other factors. Thus, the consumer choice model can also be expressed as:

(3) $$Y_{nit}^{}=\alpha X_{n}+\varepsilon_{n}$$

In the MVP model, $Y_{nit}$ is an i-dimensional column vector matrix: $Y_{nit}^{}={\left(Y_{n1t}^{},Y_{n2t}^{},......,Y_{nit}^{}\right)}^{\prime }$, and $X_{n}=\left[\begin{array}{cccc} X_{n11}\ldots X_{n1k} & & & \\ & X_{n21}\ldots X_{n2k} & & \\ & & ...... & \\ & & & X_{ni1}\ldots X_{nik} \end{array}\right]$ is a i×(i×k) dimensional quasi-diagonal matrix, where $X_{nik}$ is the independent variable *k* that affects participant *n* to choose type *i* information attribute in the MBC experiment. In Equation (3), α is a parameter matrix to be estimated: $\alpha =\left(\alpha_{11},\alpha_{12},\ldots \alpha_{1k},\alpha_{21},\alpha_{22},\ldots \alpha_{2k},......\alpha_{n1},\alpha_{n2},\ldots \alpha_{nk},\right)$. If $\varepsilon_{ni}$ satisfies a multivariate normal distribution, MVN(0,Ψ), that follows the zero condition mean and variance values, the assumption of the MVP model is satisfied. Then, the conditional probability of participants choosing the attribute is given by

(4) $$\mathrm{Pr}ob(Y_{ni}^{}=1)=1-\Phi (-X_{ni}^{}\beta )=\Phi (X_{ni}^{}\beta )$$

The covariance matrix is as follows,

(5) $$\left[\begin{array}{cccc} 1 & \rho_{12} & \rho_{13} & \rho_{14}\\ \rho_{12} & 1 & \rho_{23} & \rho_{24}\\ \rho_{13} & \rho_{23} & 1 & \rho_{34}\\ \rho_{14} & \rho_{24} & \rho_{34} & 1 \end{array}\right]$$

The off-diagonal elements in Equation (5) represent the unobservable relation between the stochastic terms of four equations of four information attribute choice behaviors. ρ≠0 indicates that there is a correlation between the stochastic terms of each equation, and the multivariate probit model should be used for estimation.

#### 2.6.2. Variable Settings

In this study, the “supply chain traceability” attribute, “supply chain–internal traceability” attribute, “pork quality inspection” attribute, and ”quality management system certification” attribute were defined as the dependent variables Y_1_, Y_2_, Y_3_, and Y_4_, respectively. The settings and definitions of the dependent and independent variables are shown in Table 5.

## 3. Results and Discussion

### 3.1. Descriptive Statistics

#### 3.1.1. Demographic Profile

The demographics of the 345 experiment participants in this study are shown in Table 6. Overall, 50.4% of the participants were women, and the male to female ratio was 0.98. Participants’ family size was mostly three, with an average household population of 3.23. This is basically consistent with the basic census data of the Wuxi Statistics Bureau in 2015 (the Wuxi Statistical Yearbook 2015 showed that 49.5% of the population in Wuxi was male and 50.5% was female in 2014; the average household population in Wuxi was 3.06.), indicating that the sample is representative. A total of 49.3% of participants were aged 26–40 years, 40.3% had junior college or undergraduate education, and 59.5% had a monthly family income of 7000–13,000 yuan. In addition, 66.4% of the participants expressed concern about food safety and 45.5% of the participants experienced foodborne illness. The percentages of respondents who chose “totally believe” and “mostly believe” for food safety labels were 7.0% and 51.2%, respectively.

#### 3.1.2. Frequencies of Choice for Each Attribute in Menu-Based Experiment

As shown in Table 7, pork quality inspection was most frequently chosen, followed by quality management system certification, supply chain–internal traceability, and supply chain traceability. Obviously, the frequency of choice was higher for ex ante quality verification attributes than for ex post traceability attributes, which indicated that consumers preferred safety information attributes with ex ante quality verification functions. In addition, from the perspective of two functional attributes, pork quality inspection and supply chain–internal traceability were the two attributes most preferred by participants for ex ante quality verification and ex post traceability, respectively. It is worth noting that in the MBC counting analysis, although pork quality inspection had the highest average price, it still had the highest frequency of choice. This indicates that the pork quality inspection attribute was most preferred by participants. Therefore, setting the pork quality inspection attribute in the form of a label on traceable pork can more effectively convey pork safety information; it also indicates that setting the pork quality inspection attribute in the traceable pork had a certain market consumption demand.

#### 3.1.3. Relationship between the Choice of Attributes and Price

As shown in Table 8, with an increase in price level, the frequency for participants to choose supply chain traceability, supply chain–internal traceability, pork quality inspection, and quality management system certification decreased. Then, the demand price elasticity of each attribute was calculated by using the selection frequency as a substitute variable of the attribute demand. The results showed that the demand price elasticity of the supply chain traceability, supply chain–internal traceability, pork quality inspection, and enterprise quality management system certification attributes were −0.776, −1.005, −1.904, and −0.946, respectively. The demand price elasticity value of each attribute was negative, which was in line with the demand theory and also verified the negative relationship between price of the attribute itself and the possibility of the participant choosing to purchase the attribute. In addition, the elasticity value also reflected the sensitivity of the experimental participants to the attribute price. The pork quality inspection and supply chain–internal traceability attribute were all elastic attributes that were highly sensitive to price changes, while the supply chain traceability and enterprise quality management system certification attributes were all inelastic attributes that were less sensitive to price changes.

Furthermore, it should be noted that there was a negative correlation trend between the price of the supply chain traceability and the frequency of choosing supply chain–internal traceability (as shown in Table 6); after further calculation, the cross price elasticity of the supply chain traceability attribute and the supply chain–internal traceability attribute was −0.187, suggesting a complementary relationship between them. However, based on the attributes established in this study, supply chain–internal traceability was defined as the ability to obtain key historical information on internal (production) processes in farming, slaughter, transportation, and marketing, in addition to the ability to obtain relevant information provided by supply chain traceability. In terms of connotation, there is a substitute relationship between supply chain traceability and supply chain–internal traceability. The contradictions presented here may be related to the under-specification of the model. Therefore, it is necessary to further analyze the intersectional relationship between attributes based on the regression model.

### 3.2. Model Estimation and Discussion

Stata 12.0 was used to fit the multivariate probit model on the encoded data. The covariance matrix of the regression equation is shown in Table 9. The Chi-squared value of the model was equal to 501.666, and the 1% significance level test indicated that there was a correlation between the stochastic term of each equation, so employing a multivariate probit model was appropriate. In the covariance matrix, all six coefficients passed the significance test, which meant that a participant’s decision to choose an information attribute of traceable pork would be affected by whether he/she chose other information attributes of traceable pork.

The MVP model regression results of traceable pork information attribute selection behavior to participants are shown in Table 10, which indicate that the MVP model provided a good fitting result for the dataset.

#### 3.2.1. Influence of Attribute Price on the Participants’ Choice of Traceable Pork Information Attribute

(1) For the supply chain traceability attribute and supply chain–internal traceability attribute with ex post traceability, the price of “A” attribute had a significant negative influence on consumers’ choice of “A” attribute, while it had a significant positive influence on participants’ choice of supply chain–internal traceability attribute and quality management system certification attribute. This indicated that as the price of supply chain traceability attribute increased, the probability of choosing supply chain traceability attribute decreased, and participants tended to replace supply chain traceability attribute with supply chain–internal traceability attribute and quality management system certification attribute.

In addition, the price of attribute supply chain–internal traceability attribute had a significant negative impact on participants’ choice of attribute supply chain–internal traceability attribute, while a significant positive impact on participants’ choice of supply chain traceability attribute and quality management system certification attribute. This indicated that as the price of “B” increased, the probability of choosing supply chain–internal traceability attribute decreased, and participants tended to replace supply chain–internal traceability attribute with supply chain traceability attribute and quality management system certification attribute.

(2) For pork quality inspection attribute and quality management system certification attribute with ex ante quality verification, the price of pork quality inspection attribute had a significant negative influence on the choice of pork quality inspection attribute by participants, while it had a significant positive influence on the choice of quality management system certification attribute by participants. This indicated that as the price of pork quality inspection attribute increased, the probability of choosing pork quality inspection attribute by participants decreased, and participants tended to replace pork quality inspection attribute with quality management system certification attribute.

In addition, the price of quality management system certification attribute significantly negatively affected participants’ choice of quality management system certification attribute, while it had a significant positive impact on participants’ choice of pork quality inspection attribute and supply chain–internal traceability attribute. This means that as the price of quality management system certification attribute increased, the probability of choosing “D” decreased, and participants tended to choose pork quality inspection attribute and supply chain–internal traceability attribute instead of quality management system certification attribute.

(3) Therefore, attribute price was an important factor influencing consumers’ choice of information attribute. In addition, there was a two-way substitution relationship between the two attributes with ex ante quality verification, between the two attributes with ex post traceability, and between the quality management system certification attribute with ex ante quality verification and the attribute supply chain–internal traceability attribute with ex post traceability. As a consequence, under budget constraints, it is not necessary to provide consumers with traceable pork that contains four attributes in its entirety. If customization cannot be achieved, the market options that can be selected to reduce food safety risks are as follows. First, according to the counting analysis results, a profile composed of pork quality inspection and supply chain–internal traceability was the most preferred type of traceable pork in the market. Second, when the price of the pork quality inspection attribute rises, the quality management system certification attribute can be used to substitute the pork quality inspection attribute, and the profile composed of quality management system certification and supply chain–internal traceability can be one of the alternatives to the most preferred type. Third, when the price of the supply chain–internal traceability attribute rises, the profile composed of pork quality inspection and supply chain traceability can be other alternatives to the most preferred type.

#### 3.2.2. Influence of Other Factors on the Participants’ Choice of Traceable Pork Information Attribute

Heterogeneity was observed in participants’ choice of traceable pork information attribute. For example, male, older, lower-educated, and lower monthly household income consumers were more likely to choose the supply chain traceability attribute; female, higher monthly household participants were more likely to choose supply chain–internal traceability attribute and quality management system certification attribute. Young participants were more likely to choose the supply chain–internal traceability attribute and quality management system certification attribute. In addition, participants who were less concerned about food safety were more likely to choose the supply chain–internal traceability attribute. Participants with foodborne illness experience had a higher probability of choosing the supply chain traceability attribute and the pork quality inspection attribute, while participants without foodborne illness experience were more likely to choose supply chain–internal traceability attribute. Participants with higher trust in food safety labels were more likely to choose supply chain–internal traceability attribute and pork quality inspection attribute, while participants with lower trust in food safety labels were more likely to choose supply chain traceability attribute.

## 4. Conclusions

In this study, two ex ante quality verification attributes, pork quality inspection and quality management system certification, and two ex post traceability attributes, supply chain traceability and supply chain–internal traceability, were established for traceable pork. Interactions between safety information attributes and the consumer’s response to cost-driven price changes were investigated for 345 consumers in Wuxi, China, using the menu-based choice (MBC) experiment and the multivariate probit (MVP) model. Results showed that, first, food safety information attributes were important to consumers. Compared with the ex post traceability attributes, the consumers preferred safety information attributes with an ex ante quality verification function. In the ex ante quality verification attributes, the consumer’s preference for the pork quality inspection attribute was superior to the quality management system certification attribute. Therefore, setting the pork quality inspection attribute in the form of a label on traceable pork can more effectively convey pork safety information. It also indicated that setting the pork quality inspection attribute for the traceable pork had a certain market consumption demand.

Second, pork quality inspection and supply chain–internal traceability were the two elastic attributes most preferred by consumers for ex ante quality verification and ex post traceability, respectively. When customization cannot be achieved or subject to budget constraint, a profile composed of pork quality inspection and supply chain–internal traceability would be the most preferred traceable pork product in the market based on the need of building a fully functional traceable food system and reducing food safety risks. Third, attribute price was an important factor that affected consumers’ choice of information attribute. The relationship between attribute price and demand indicated that there was a strong substitution relationship between different information attributes. In addition, heterogeneity was observed in consumers’ choice of traceable pork information attribute. Individual and family characteristics, foodborne illness experiences, food safety concerns, and trust in safety information labels all significantly affected consumers’ choice of information attributes. Fourth, the pork quality inspection attribute and supply chain–internal traceability attribute are attributes with a greater elasticity of demand. If the government can subsidize traceable pork consisting of these two attributes, it will not only increase consumer demand but also increase the profitability of the relevant traceable pork suppliers, which may motivate traceable pork suppliers to proactively provide quality and safety information.

This research provides guidelines for promoting the development of a safe food market system and the reduction of food safety risks, as well as foodborne diseases, in China. The government should encourage manufacturers to produce traceable food with food quality inspection via customization through subsidies and other policies to meet consumer demand for safe food and to reduce the spread of foodborne diseases. In addition, as supply chain traceability is a basic requirement of consumers, ex post traceability should cover all the risk processes of the entire food supply chain. Furthermore, the government should support manufacturers in producing multi-level safe food to meet diverse consumer demand and gradually promote the construction of a traceable food market system, and manufacturers should dynamically adjust their production and marketing strategies for different types of safe food based on consumer preferences. This study has some limitations. For example, a possible explanation for the complexity of the aggregate price effect exhibited by the Marshallian demand function in different market situations in this paper only was conducted on the theoretical level, and there was a lack of statistical test tools to further verify this theoretical interpretation. In addition, for the sake of simplicity, this study did not conduct a market simulation based on the scenario that ordinary pork and safe pork with different attributes and level combinations are circulated in the market simultaneously. Therefore, future studies should overcome this limitation and propose development pathways for China’s food safety system that are in accordance with the actual situation in the country.

## Figures and Tables

**Table 1:** Attributes and price levels of traceable pork with different functions (price: yuan/500 g).

Function	Attribute	Description of Attribute
Ex post traceability	Supply chain traceability	Scanning type I barcode through a public inquiry platform, consumers can obtain the basic information about enterprise in the pork supply chain.
	Supply chain–internal traceability	Scanning type II barcode through a public inquiry platform, consumers can obtain information about what type I has, as well as critical internal safety records such as the source of feed, veterinary drug use, and inspection.
Ex ante quality verification	Pork quality inspection	Labeling products as acceptable after testing of physical and chemical indicators, such as pesticide and veterinary drug residues, and microbial indicators, such as number of *Escherichia coli*, by a qualified testing agency (as feed may contain pesticide residues and thereby contaminate pork, pesticide residues should be included as one of the physical and chemical indicators in pork quality inspection).
	Quality management system certification	Labeling slaughtering and processing enterprises with a mark of quality management system certification after review of quality and safety management capability by a qualified certification agency.

Note: As feed may contain pesticide residues and thereby contaminate the pork, pesticide residues should be included as one of the physical and chemical indicators in pork quality inspection.

**Table 2:** Consumer maximum premium for different information attributes (price: yuan/500 g).

Attribute	Max.	Min.	Mean	Standard Deviation
Supply chain traceability	10.0	0.0	2.9	1.6
Supply chain–internal traceability	10.0	0.0	3.4	1.8
Pork quality inspection	11.0	0.0	3.9	2.1
Quality management system certification	9.0	0.0	3.2	1.9

**Table 3:** Price levels of traceable pork attributes with different functions (price: yuan/500 g).

Price Level Setting	Attributes with Ex Post Traceability Function		Attributes with Ex Ante Quality Verification Function	
	Supply Chain Traceability	Supply Chain–Internal Traceability	Pork Quality Inspection	Quality Management System Certification
Price Level 1	Price 1 = 1.3	Price 1 = 1.6	Price 1 = 1.8	Price 1 = 1.3
Price Level 2	Price 2 = 2.1	Price 2 = 2.5	Price 2 = 2.9	Price 2 = 2.3
Price Level 3	Price 3 = 2.9	Price 3 = 3.4	Price 3 = 3.9	Price 3 = 3.2
Price Level 4	Price 4 = 3.7	Price 4 = 4.3	Price 4 = 5.0	Price 4 = 4.2
Price Level 5	Price 5 = 4.5	Price 5 = 5.2	Price 5 = 6.0	Price 5 = 5.1

**Table 4:** Efficiency test results of the attributes and levels design.

Attribute	Level	Freq.	Actual	Ideal	Effic.
Supply chain traceability	Price Level 1	22	-	-	-
	Price Level 2	21	0.2921	0.3215	0.9087
	Price Level 3	20	0.2897	0.3215	0.9011
	Price Level 4	20	0.2847	0.3215	0.8856
	Price Level 5	17	0.2691	0.3215	0.8371
Supply chain–internal traceability	Price Level 1	16	-	-	-
	Price Level 2	20	0.2888	0.3215	0.8982
	Price Level 3	21	0.2744	0.3215	0.8534
	Price Level 4	21	0.2657	0.3215	0.8263
	Price Level 5	22	0.2675	0.3215	0.8319
Pork quality inspection	Price Level 1	21	-	-	-
	Price Level 2	20	0.3226	0.3203	0.9852
	Price Level 3	19	0.3277	0.3203	0.9553
	Price Level 4	21	0.3144	0.3203	1.0378
	Price Level 5	19	0.3230	0.3203	0.9833
Quality management system certification	Price Level 1	20	-	-	-
	Price Level 2	20	0.3228	0.3190	0.9766
	Price Level 3	20	0.3265	0.3190	0.9547
	Price Level 4	20	0.3229	0.3190	0.9761
	Price Level 5	20	0.3254	0.3190	0.9615

Note: Ideal denotes the standard deviation that meets the orthogonality condition. Effic. is the ratio of actual to ideal standard deviation under the assumption that the sample size is the same for each version.

**Table 5:** Settings and definitions of the variables.

Variable	Definition
Dependent variables	
Supply chain traceability attribute (Y_1_)	Select this attribute = 1, otherwise = 0
Supply chain–internal traceability attribute (Y_2_)	Select this attribute = 1, otherwise = 0
Pork quality inspection attribute (Y_3_)	Select this attribute = 1, otherwise = 0
Quality management system certification attribute (Y_4_)	Select this attribute = 1, otherwise = 0
Independent variable: key explanatory variables	
Price of supply chain traceability attribute (X_1_)	Price
Price of supply chain–internal traceability attribute (X_2_)	Price
Price of pork quality inspection attribute (X_3_)	Price
Price of quality management system certification attribute (X_4_)	Price
Independent variable: control variables	
Sex (X_5_)	Male = 1, Female = 0
Age (X_6_)	Years
Degree (X_7_)	Primary school and below = 1; Junior high school = 2; High school = 3; Junior college = 4 Bachelor = 5; Master and above = 6
Monthly family income (X_8_)	3000 yuan and below = 1; 3000–4999 yuan = 2; 5000–6999 yuan = 3; 7000–8999 yuan = 4; 9000–10,999 yuan = 5; 11,000–12,999 yuan = 6; 13,000–14,999 yuan = 7; 15,000–17,999 yuan = 8; 18,000 yuan and above = 9
Concern about food safety (X_9_)	Totally unconcerned = 1; More unconcerned = 2; 50% Concerned = 3; More concerned = 4; Very concerned = 5
Foodborne illness experience (X_10_)	Yes = 1, No = 0
Believe in food safety labels (X_11_)	Totally believe = 1; Mostly believe = 2; 50% Believe = 3; Mostly disbelieve = 4; Do not believe at all = 5

**Table 6:** Sociodemographic statistics.

Demographics	Classification Index	Sample Size	Percent %
Gender	Male	171	49.6
	Female	174	50.4
Age	25 years or younger	67	19.4
	26–40 years	170	49.3
	41–60 years	99	28.7
	61 years or older	9	2.6
Education	Junior high school or lower	154	44.6
	Junior college or undergraduate	139	40.3
	Postgraduate or above	52	15.1
Family Size	1 people	5	1.5
	2 people	21	6.1
	3 people	155	44.9
	4 people	77	22.3
	5 people or more	87	25.2
Monthly Family Income	3000 yuan or less	3	0.9
	3000–4999 yuan	6	1.7
	5000–6999 yuan	44	12.7
	7000–8999 yuan	83	24.0
	9000–10,999 yuan	72	20.9
	11,000–12,999 yuan	50	14.5
	13,000–14,999 yuan	34	9.9
	15,000–17,999 yuan	21	6.1
	18,000 yuan or more	32	9.3
Concern about food safety	Totally unconcerned	2	0.6
	More unconcerned	17	4.9
	50% concerned	97	28.1
	More concerned	169	49.0
	Very concerned	60	17.4
Have foodborne illness experience	Yes	157	45.5
	No	188	54.5
Believe in food safety labels	Totally believe	24	7.0
	Mostly believe	177	51.2
	50% believe	109	31.6
	Mostly disbelieve	22	9.6
	Do not believe at all	2	0.6

**Table 7:** Number and frequency of the choice for each attribute in the menu-based choice experiment.

Attribute	Chosen or Not	Number of Choices	Frequency of Choice (%)
Supply chain traceability	Yes	438	12.7
Supply chain–internal traceability	Yes	909	26.4
Pork quality inspection	Yes	1086	31.5
Quality management system certification	Yes	931	27.0

**Table 8:** Frequency of choice for each attribute at different price levels.

Price Category	Price Value	Supply Chain Traceability (%)	Supply Chain–Internal Traceability (%)	Pork Quality Inspection (%)	Quality Management System Certification (%)
Price of supply chain traceability	Ұ1.3	16.60	42.80	27.10	27.30
	Ұ2.1	15.40	27.60	28.30	21.10
	Ұ2.9	14.00	20.20	31.60	28.30
	Ұ3.7	7.40	17.70	39.90	29.60
	Ұ4.5	6.70	17.10	32.80	30.50
Price of supply chain–internal traceability	Ұ1.6	12.40	50.50	27.10	30.00
	Ұ2.5	14.40	35.40	28.80	23.00
	Ұ3.4	14.00	23.80	31.20	28.30
	Ұ4.3	15.20	19.60	34.00	25.60
	Ұ5.2	8.30	14.40	34.00	28.30
Price of pork quality inspection	Ұ1.8	11.50	23.80	53.90	25.90
	Ұ2.9	11.10	25.90	43.30	23.70
	Ұ3.9	13.20	29.80	25.60	25.30
	Ұ5.0	14.80	27.50	19.10	29.70
	Ұ6.0	12.90	24.80	15.40	30.30
Price of quality management system certification	Ұ1.3	10.40	28.30	27.10	46.70
	Ұ2.3	12.20	22.30	31.00	33.90
	Ұ3.2	12.90	25.60	31.20	24.80
	Ұ4.2	14.50	24.40	36.40	16.70
	Ұ5.1	13.50	31.55	31.40	12.90

**Table 9:** The covariance matrix of the multivariate probit model.

	Choose Supply Chain Traceability Attribute	Choose Supply Chain–Internal Traceability Attribute	Choose Pork Quality Inspection Attribute	Choose Quality Management System Certification Attribute
Choose supply chain traceability attribute	--	--	--	--
Choose supply chain–internal traceability attribute	−0.412 *** (0.030)	--	--	--
Choose pork quality inspection attribute	−0.201 *** (0.031)	−0.192 *** (0.029)	--	--
Choose quality management system certification attribute	−0.225 *** (0.032)	−0.158 *** (0.031)	−0.198 *** (0.030)	--
Chi-squared value	501.666			
Significance level	0.000			
Likelihood ratio test	$\rho_{21}$ = $\rho_{31}$ = $\rho_{41}$ = $\rho_{32}$ = $\rho_{42}$ = $\rho_{43}$ = 0			

Note: *** denote significance at the 1% levels.

**Table 10:** Multivariate probit (MVP) model estimation results for each attribute choice.

Dependent Variable	Model 1: Choose Supply Chain Traceability Attribute (Y_1_)	Model 2: Choose Supply Chain–Internal Traceability Attribute (Y_2_)	Model 3: Choose Pork Quality Inspection Attribute (Y_3_)	Model 4: Choose Quality Management System Certification Attribute (Y_4_)
Key explanatory variables				
Price of supply chain traceability (X_1_)	−0.313 *** (0.037)	0.089 ** (0.036)	0.040 (0.033)	0.116 *** (0.035)
Price of supply chain–internal traceability (X_2_)	0.205 *** (0.035)	−0.344 *** (0.035)	0.016 (0.033)	0.085*** (0.035)
Price of pork quality inspection (X_3_)	0.009 (0.020)	0.011 (0.017)	−0.301 *** (0.018)	0.046 *** (0.017)
Price of quality management system certification (X_4_)	0.030 (0.020)	0.037 ** (0.017)	0.041** (0.017)	−0.275 *** (0.018)
Control variables				
Sex (X_5_)	0.257 *** (0.055)	−0.127 *** (0.049)	−0.124 *** (0.048)	0.018 (0.048)
Age (X_6_)	0.007 ** (0.003)	−0.014 *** (0.002)	−0.001 (0.002)	−0.013 *** (0.003)
Degree (X_7_)	−0.006 (0.024)	0.017 (0.021)	−0.043 ** (0.020)	0.062 *** (0.020)
Monthly family income (X_8_)	−0.027 * (0.015)	0.033 ** (0.014)	0.134 *** (0.014)	−0.005 (0.014)
Concern about food safety (X_9_)	−0.010 (0.033)	−0.104 *** (0.030)	−0.023 (0.030)	0.042 (0.031)
Have foodborne illness experience (X_10_)	−0.167 ***(0.057)	0.115 **(0.050)	−0.196 ***(0.049)	−0.062(0.050)
Believe in food safety labels (X_11_)	0.097 *** (0.036)	−0.185 *** (0.030)	−0.040 (0.030)	−0.069 ** (0.031)
ASC constant	−1.276 *** (0.271)	1.020 *** (0.238)	0.103 (0.230)	0.325 (0.237)

Note: The number in brackets is standard error. *, **, and *** denote significance at the 10%, 5%, and 1% levels, respectively. ASC is alternative specific constant. Log likelihood = −6565.7754; Wald Chi-squared (44) = 1169.80; Prob > Chi-sqaured = 0.0000. All results are robust regression results (the mvprobit command in Stata12.0 was used for multivariate probit model estimation. If the robust option was added after the command, then robust regression results were obtained).

## Appendix A

### Appendix A.1. The Preparation of the BDM Auction Experiment

Before the start of the experiment, each participant was given 0.5 kg of regular pork and 5.0 yuan in cash as an incentive to participate in the experiment. Participants were told that the price of regular pork was 14.0 yuan/0.5 kg in the city where the experiment was carried out. Next, four types of traceable pork for auction were shown to the participants, who were told that the four types of traceable pork were not different from regular pork in packaging, appearance, weight, or any other visible features, except for quality and safety risks. The experiment conductors explained the inspection and certification marks. Then, participants were shown how to obtain traceability information by a computer-based traceability code search system. This was done to ensure that participants had full confidence in the authenticity of the items for auction, to minimize measurement errors resulting from non-confidence.

### Appendix A.2. The Procedure of the BDM Auction Experiment

The experiment conductors first asked the bidders to submit a bid, representing the maximum premium they were willing to pay for each type of pork with information attributes. Then, the experiment conductors gained a random price generated from the computer’s generator program (the randomly extracted binding value was unknown by the bidders) and compared the bidder’s maximum premium and the randomly generated price from our computer. The bidders won the bid if his/her maximum premium was larger than the computer-generated one; otherwise, their bids failed. After four rounds of bidding, the computer program randomly picked one set of scores from the four rounds of bidding results. For that randomly chosen set of scores, if the bidder won the bidding, then the experiment conductors exchanged the corresponding type of safe pork and payed a price that was randomly generated by the computer; otherwise, the bidder could not exchange for the pork with higher safety attributes and could only keep the previously given ordinary pork.

### Appendix A.3. A Further Discussion on the Results of the BDM Auction Experiment

According to the modeling of preference-payment behavior [32], it is assumed that a consumer’s WTP reflects their preference rankings for the attributes. Table 2 in the paper shows that the attributes, in descending order of preference, were pork quality inspection, supply chain–internal traceability, quality management system certification, and supply chain traceability. Obviously, the rankings of the four attribute preferences indicated by the BDM experiment were different from the rankings of the MBC experiment. However, the experimental results shown in the BDM and MBC experiments demonstrated that the pork quality inspection attribute was most preferred by participants. In addition, pork quality inspection and supply chain–internal traceability were the two attributes most preferred by participants for ex ante quality verification and ex post traceability, respectively. However, due to sample size limitations, only a single attribute was auctioned in the BDM auction, and the possible substitutes or complementary relationships between the attributes were not evaluated. By contrast, the MBC experiment allowed us to effectively determine the interrelationships between the attributes, which were further confirmed by the MBC experimental results.
